# Enhanced Visual Feedback Using Immersive VR Affects Decision Making Regarding Hand Use With a Simulated Impaired Limb

**DOI:** 10.3389/fnhum.2021.677578

**Published:** 2021-06-11

**Authors:** Naoko Sakabe, Samirah Altukhaim, Yoshikatsu Hayashi, Takeshi Sakurada, Shiro Yano, Toshiyuki Kondo

**Affiliations:** ^1^Department of Computer and Information Sciences, Graduate School of Engineering, Tokyo University of Agriculture and Technology, Tokyo, Japan; ^2^Biomedical Science and Biomedical Engineering, School of Biological Sciences, University of Reading, Reading, United Kingdom; ^3^Physiotherapy Group in Stroke Unit, Alamiri Hospital, Kuwait City, Kuwait; ^4^College of Science and Engineering, Ritsumeikan University, Shiga, Japan

**Keywords:** immersive virtual reality, learned non-use, constraint-induced movement therapy, reinforcement-induced movement therapy, visual amplification

## Abstract

The long-term effects of impairment have a negative impact on the quality of life of stroke patients in terms of not using the affected limb even after some recovery (i.e., learned non-use). Immersive virtual reality (IVR) has been introduced as a new approach for the treatment of stroke rehabilitation. We propose an IVR-based therapeutic approach to incorporate positive reinforcement components in motor coordination as opposed to constraint-induced movement therapy (CIMT). This study aimed to investigate the effect of IVR-reinforced physical therapy that incorporates positive reinforcement components in motor coordination. To simulate affected upper limb function loss in patients, a wrist weight was attached to the dominant hand of participant. Participants were asked to choose their right or left hand to reach toward a randomly allocated target. The movement of the virtual image of the upper limb was reinforced by visual feedback to participants, that is, the participants perceived their motor coordination as if their upper limb was moving to a greater degree than what was occurring in everyday life. We found that the use of the simulated affected limb was increased after the visual feedback enhancement intervention, and importantly, the effect was maintained even after gradual withdrawal of the visual amplification. The results suggest that positive reinforcement within the IVR could induce an effect on decision making in hand usage.

## 1. Introduction

Stroke is one of the leading causes of long-term disability, and it has a higher prevalence in older people. It has been reported that the number of stroke survivors is increasing in the aging society (Mukherjee and Patil, 2011). Stroke patients may suffer from upper limb impairment and experience many of challenges while undergoing treatment for this impairment (Adamovich et al., 2004), and often such treatment requires several different approaches (Dobkin, 2008). There is great variability in terms of functional outcome; some patients are unable to regain full functionality, and they must manage with varying levels of lifelong upper limb paresis (Faria-Fortini et al., 2011). Therefore, there is a need to develop rehabilitation methods that can offer more effective treatment and a better chance of higher-level functional recovery.

One of the most appreciated rehabilitation processes is motor coordination (MC), which plays a vital role in the musculoskeletal system, and leads to movement of body parts (Levin et al., 2008). Motor coordination is facilitated by the forces of kinetic features and kinematic actions that work together to achieve voluntary movements (Arbib, 1981). For most of stroke patients, rehabilitation primarily involves physical therapy (PT). The foremost role of PT is to achieve coordination of the affected parts of the body. This can be investigated in depth by discussing the role of neuroplasticity in the rehabilitation of neurons of stroke patients (Mang et al., 2013). Physiotherapy sessions mainly involve task-oriented exercises that can restore healthy activities such as pouring water into a glass and drinking or reacquiring skills (Rensink et al., 2009; Ordahan et al., 2015).

Constraint-Induced Movement Therapy (CIMT) is one of the most effective treatment procedures for stroke patients. This method involves physical constraining of unaffected limb and compelling the patient to use the affected limb. Based on previous studies, a noticeable improvement is observed in experimental patients as compared to other therapies (Kwakkel et al., 2015). Although this method is successful, researchers have argued that CIMT processes are often intensive and involve a grueling therapeutic schedule that may affect a patient's adherence to a therapy regimen, which affects the efficacy of the treatment (Lannin et al., 2003; Kwakkel et al., 2015). Virtual reality (VR) has shown promising results in terms of recovery of stroke patients and clinical feasibility (Burdea, 2003; Perez-Marcos et al., 2017). The use of VR technology allows for the use of more dynamic environmental setups in which targets can be reliably and rapidly modified (Viau et al., 2004).

Immersive virtual reality (IVR) continues to show great promise when applied to CIMT as part of post-stroke rehabilitation. Numerous studies have shown that IVR rehabilitation could greatly improve affordability and accessibility of post-stroke motor treatment in an immersive and interesting way, while also providing incredibly valuable, individualized bio-feedback to therapists to improve the rehabilitation process.

The IVR environment significantly improves patient motivation to perform repetitive, motor-intensive tasks that are a crucial component of the rehabilitation process (Elor et al., 2018a,b). It was shown how a simple game with adaptive difficulty could lead to improved mobility, motor performance, and psychological health when compared to standard rehabilitation techniques. Elor et al. used IVR incorporating modified constraint-induced movement therapy (mCIMT), and the results showed that the proposing system was beneficial for stroke patients by encouraging them to use the affected hand without constraining the unaffected side (Both hands are free to move). Another study aims to determine the feasibility and measure safety and outcomes of combining IVR and mCIMT (Borstad et al., 2018). They used the “Recovery Rapids” kayaking game in the Virtual Environment to provide a motivating environment. From the outcome measures, the combination between IVR and mCIMT could be greatly improved for patients with chronic hemiparesis. The patients were much more likely to engage with rehabilitation when done in this sort of engaging and interactive way (Borstad et al., 2018). In regards to implementation, another study showed that a head-mounted display (HMD) was a much more effective tool for physiological rehabilitation and encouraging task-based exercise than a dedicated interactive room environment which projected the games onto the four walls of the room (Elor and Kurniawan, 2020; Elor et al., 2020). This is because the HMD-based system was perceived to have a higher sense of immersion, ease of use, and enjoyment of gameplay than the room-scale alternative.

As a direct relevance to our study, Ballester et al. (2016) offered a new treatment alternative that incorporates CIMT and another therapeutic approach known as reinforcement induced movement therapy (RIMT). Using the goal-oriented reaching task, they amplified the speed of the impaired hand within the VR environment and showed the efficacy of the RIMT. Even though the improvement of the motor coordination of stroke patients was demonstrated in the Fugle-Mayer scores after the RIMT intervention, the subjective experience of being fully immersed in the VR environment was not realized in their study. Although they introduced positive reinforcement in their pioneering work using computer simulated limb in the display, the concept of the RIMT should be extended to use the Immersive VR. In IVR, visual feedback is set up to show only the simulated upper limb. It is important that participants observe only the simulated hand during the task. If they observe their real hand moving in front of them while watching the simulated hand on the display, the mismatch in visual feedback of the motor coordination would create a sense of loss of ownership of the simulated upper limb, or, in some cases, the subjective awareness of loss of controlling one's own body.

The learning process entails the creation of new connections. This process is made possible by neuroplasticity (Mang et al., 2013). Task-oriented training increases activation in parts of the brain, such as the inferior parietal cortex, the premotor cortex, and the sensorimotor cortex; therefore increasing neuroplasticity in the motor and sensory neural pathways (Nelles et al., 2001; Jang et al., 2003; Rensink et al., 2009). In addition, body representation in a coordinated system of the body based on the visual information. This plays a critical role in the subjective experience of motor coordination in goal-oriented tasks. Thus, in this study, we explored the RIMT paradigm by using the IVR to produce the subjective experiences. This study presents a new therapeutic approach for IVR that incorporates RIMT by simulating impaired conditions in healthy participants.

## 2. Materials and Methods

### 2.1. Participants

The experiments were conducted at two sites: Tokyo University of Agriculture and Technology (TUAT), Japan and the University of Reading (UoR), UK. Seventeen healthy individuals (nine female and eight male) participated in the experiment at the TUAT, while 29 healthy individuals (17 female and 12 male) participated in the experiments at the UoR. In total, 46 healthy participants were recruited. All were right-handed, and their mean age was 27.5 ± 7.9 (mean ± sd). Participants provided written informed consent after being informed about the aims and procedure of the experiment. The experimental protocols were approved by the ethics committees of both TUAT (No.191204-3145) and UoR (No.SBS18-19 17), and the devices, programs, and protocol of participant experiments were identical at both sites.

### 2.2. Experimental Setup

**Figure 1:**
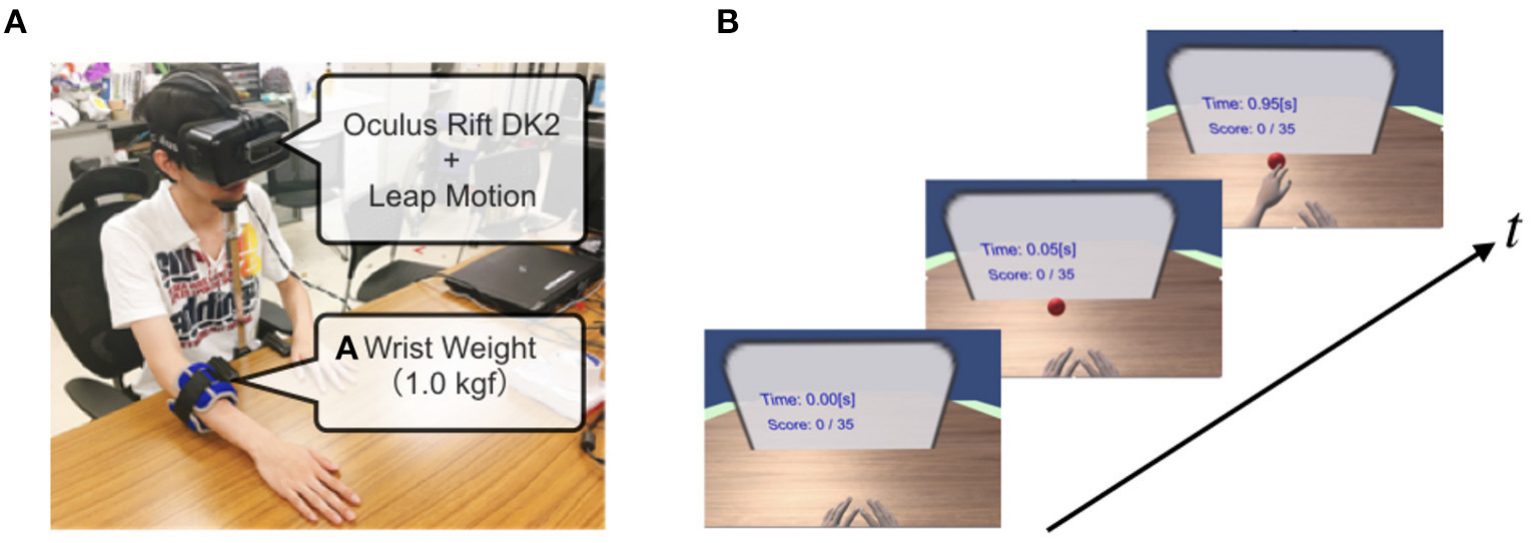
Immersive Virtual Reality system integrated with motion capture. **(A)** A 1.0 kg wrist weight was attached to the distal portion of the dominant forearm of the healthy participant to simulate the affected upper limb of stroke patients. A head-mounted display (HMD) was attached to the head with a strap, and a motion capture system (Leap motion) was attached to the front part of the HMD. **(B)** The participant placed their hands at the home positions, and the target appeared randomly along with the semi-circle. The participant was asked to reach for the target immediately by choosing their virtually impaired or unaffected hand.

We developed an IVR system (Figure 1A) integrated with a HMD (Oculus Rift DK2) and a markerless motion capture system (Leap Motion). The motion capture device was fixed on the front of the HMD to measure the real hands movements in the frontal space. Participants were asked to wear the HMD, and sit comfortably on a high-back chair and rest their upper limbs on a table placed in front of them. Through the IVR system, the participants could experience a fully embodied avatar, and the virtual hand was controlled to coincide with the captured real hand position in real-time using the Leap Motion Core Assets (version: 4.3.2; Figure 1B). The VR environment and virtual upper limbs were implemented using Unity (version: Unity2018.2.9) and Blender (version: 2.79b) software. The virtual upper limb posture (i.e., the shoulder and elbow angles) in the IVR environment was adjusted by an inverse kinematics program (Final IK, version: 1.8) in the Unity environment. Thus, participants were able to execute the reaching task in the IVR environment from the first-person perspective.

To realize visual enhancement intervention, we introduced a visual amplification function in the IVR system, in which the virtual hand was displayed at α times linearly apart, that is, the position vector of the virtual hand starting from a home position was extended α times with respect to the actual hand position vector. Even in the visual enhancement condition, the appearance of the virtual upper limb posture was adjusted by the inverse kinematics program.

### 2.3. Task

**Figure 2:**
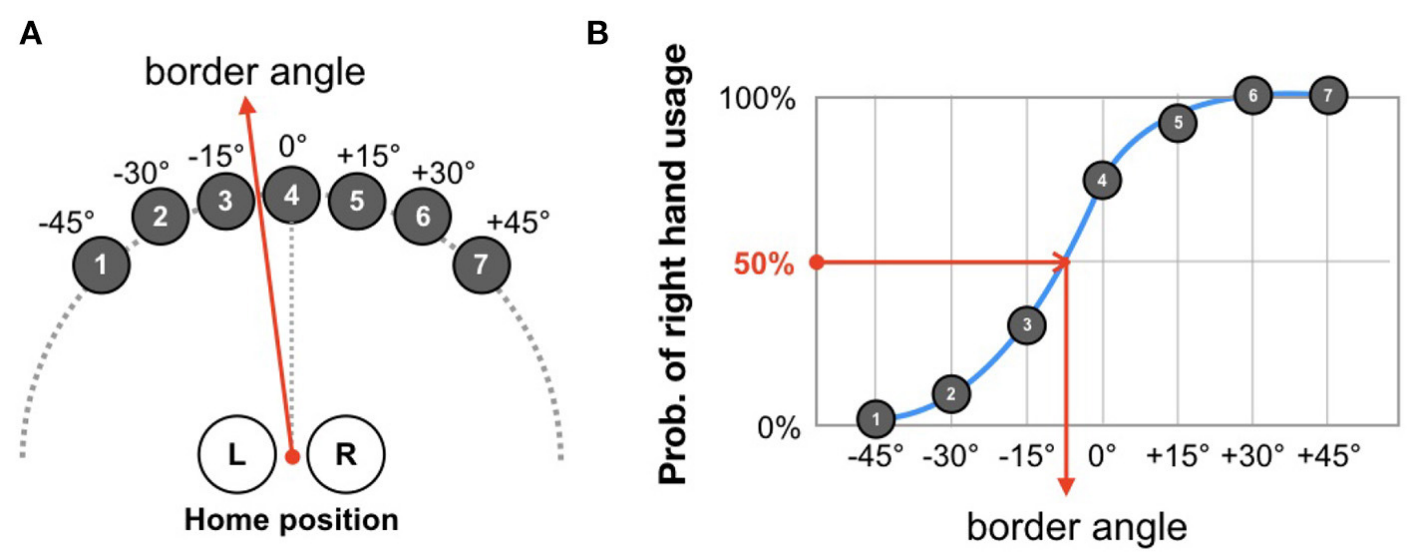
Definition of border angle. **(A)** At each trial, participants were asked to reach toward a target that was randomly drawn from seven candidate positions. The task was repeated until 70 trials were completed (10 times for each target). **(B)** According to the probability distribution of right-hand usage, the border angle is determined as the angle-approximated psychometric function that results in a probability of 50%.

In the VR environment, the participants engaged in a hand-choice reaching task (Figure 2A). At the beginning of each trial, participants were instructed to set both virtual hands at a home position. A half-second after the initialization, a virtual target (red sphere) randomly appeared in one of seven candidate positions (0, ±15, ±30, and ±45° from midline). They were asked to reach for the target immediately by choosing their right or left hand. The target turned in blue when reached by the virtual hand and immediately disappeared. To prevent in-depth consideration for choice, the experimental system checked that the reaction time was between 150 and 500 ms after the appearance. If this condition is not fulfilled, the target will disappear and the trial is invalidated.

To investigate whether the enhanced visual feedback in the IVR system affects decision making in choice of hand use among impaired persons, we introduced a virtual impairment condition aimed at simulating stroke patients with degradation of upper limb function. We attached a heavy wrist weight (1.0 kg) to the distal portion of the dominant forearm of the healthy participants. To determine the mass of the wrist weight, we performed a pilot study. The weight of 0.5 kg did not affect the usage of the hand (i.e., hand choice), but the weight of 1.0 kg showed the effect of the weight to simulate the impaired upper limb.

### 2.4. Procedure

**Figure 3:**
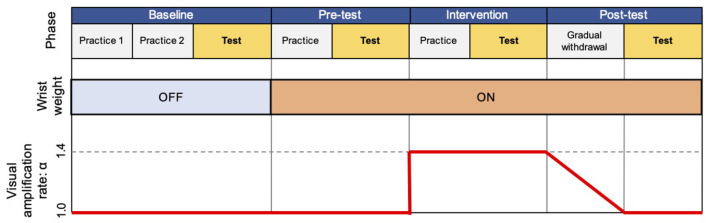
Flow of experiment. The experiment consisted of four experimental phases (baseline, pre-test, intervention, and post-test), each of which includes practice or gradual withdrawal session, and a test session.

The experiment consisted of four phases: baseline, pre-test, Intervention, and post-test (Figure 3).

The baseline phase aimed to familiarize participants with the task procedure and measure their baseline performance, including two practice sessions and one test session. In the first practice session, we asked participants to perform the reaching task using their right hand. Each target appeared twice in a fixed order (left to right). Thereafter, they repeated the same procedure with their left hand. Through this practice session, participants could perform the reaching task toward all the target with each hand. In the second practice session, participants were instructed to reach their hand to a randomly appearing target by freely choosing the right or left hand. This was repeated 5 times for each target. After these practice sessions, participants completed a total of 70 trials (10 times per target) with randomly appearing targets as the test session. Results from the test session were used as the baseline performance for the free choice reaching paradigm without wrist weight and visual amplification.

The pre-test phase included one practice and one test session with wrist weight, but no visual amplification, and aimed to quantify the effect of the attached wrist weight in choosing their right or left hand. The practice session contained 35 trials, that is, 5 times for each target, and was introduced to endow the participants with the experience of the reaching task with the wrist weight. After the practice session, participants executed a test session following the same protocol as the test session of the baseline phase.

In the intervention phase, we repeated the same procedure as in the pre-test phase, but with the visual amplification (α = 1.4).

After the intervention phase, one gradual withdrawal session was conducted with gradually reducing the visual amplification to α = 1.0 at the end. Finally, the post-test phase was executed without visual amplification, following the same protocol as in the test session of the pre-test phase.

In each session, the participants repeated the reaching task at their own pace. It was <3 min for 70 trials. To minimize their fatigue, two minutes breaks were provided between phases. The entire procedure takes 35–40 min.

### 2.5. Analysis and Statistics

As shown in Figure 2A, targets appeared in a semi-circular array at seven predetermined angles separated by 15°. To analyze the effects of the wrist weight and the visual enhancement, we quantified participants' usage of their left and right hands in the test session of each phase. To this end, first, the probability of right hand usage was plotted as a function of the target angles, and then a psychometric function was fitted to these plots (Figure 2B). The angle at which the psychometric function corresponds to a 50% probability was defined as the border angle.

To test if the wrist weight leads to less usage of the dominant hand and visual enhancement causes recovery of the usage, the border angles during the four experimental phases were statistically evaluated using the Friedman test. For further analysis, we conducted paired comparisons of the border angle distributions in each phase. The Shapiro-Wilk test was used to check the normality of the distribution, and the Wilcoxon signed-rank test was applied to evaluate statistical significance among phases. The significance level was set to *p* = 0.05.

## 3. Results

Three out of 46 participants were excluded from further analysis for the reason that the border angle could not be calculated because they chose an inappropriate strategy, for example, they decided to use their right or left hand for all the targets in any phases.

To reveal the effects of wrist weight and visual amplification on the distributions of border angles among the four experimental phases, we performed the Friedman test for the remaining 43 participants. As a result, we confirmed statistical significance among the four phases (*F*_*r*_ = 33.795, *df* = 3, *p* < 0.01). Before the paired comparison analysis, we performed the Shapiro–Wilk test to check if the data met the normal distribution criteria. The resultant *p*-value of the normality test was *p* = 0.006251, *p* = 0.001783, *p* = 0.06966, and *p* = 0.06293 for the baseline, pre-test, intervention, and post-test phases, respectively. Since the data for the border angles in some phases were not normally distributed, we decided to use the Wilcoxon signed-rank test for the paired sample comparison among the phases.

**Figure 4:**
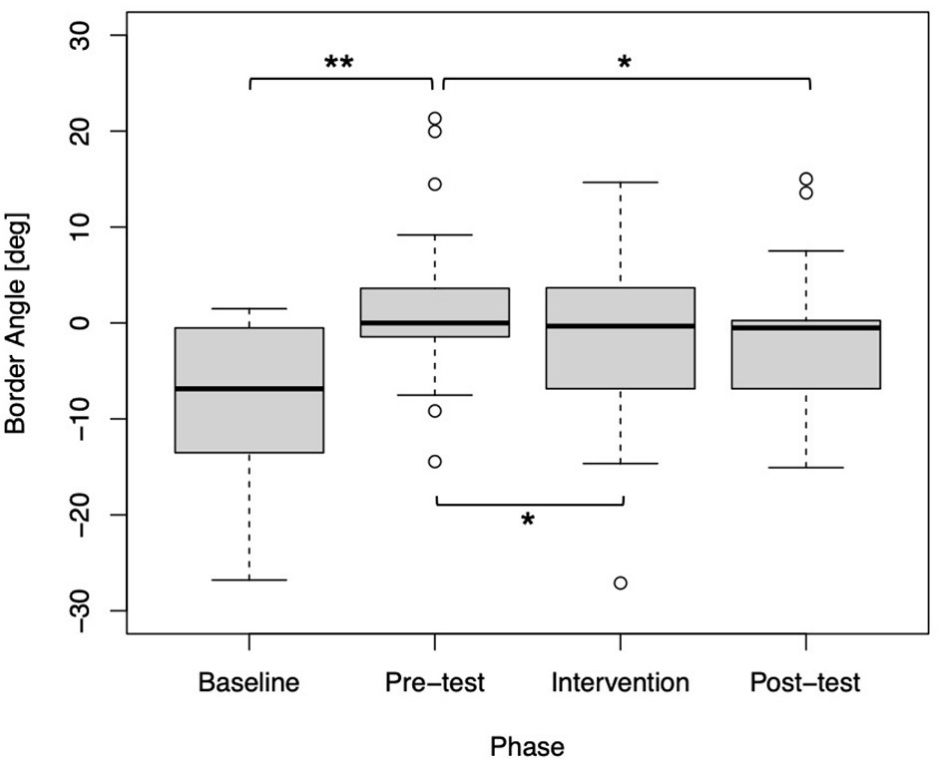
Box plot of the border angles for each phase (*N* = 43). Statistical significance was confirmed between the baseline and pre-test phases (^**^p < 0.01), the pre-test and intervention phases (^*^p < 0.05), and the pre-test and post-test phases (*p* < 0.05).

As shown in Figure 4, the statistical comparison between the baseline and pre-test phases was significant (*p* < 0.01). This suggests that attaching a wrist weight reduced the frequency of using their dominant (i.e., right) hand to reach for the target. Thus, it was a successful manipulation to simulate impaired function of a stroke patient, whereas the effect was not confirmed in six participants. Moreover, the statistical comparison between the pre-test and intervention phases was also significant (*p* = 0.01347). This implies that visual amplification affected the usage of their dominant hand when performing the task. Importantly, the result of the comparison between the pre-test and post-test phases was significant (*p* = 0.02432). This indicates that the visual amplification in the intervention phase induced a positive effect on the use of the simulated impaired hand even after the visual amplification was removed (i.e., post-test).

## 4. Discussion

This study aimed to test the validity of a new therapeutic approach that uses IVR technology. Ballester et al. (2016) offered a new treatment alternative that incorporates CIMT and another therapeutic approach known as reinforcement induced movement therapy (RIMT). Our IVR approach incorporates this positive reinforcement in physical rehabilitation as opposed to other conventional CIMT. In this study, we tested whether the visual enhancement intervention realized by the IVR system causes positive reinforcement in a hand-choice reaching task. To simulate the impaired upper limb function of stroke patients in healthy participants, we introduced a virtual impairment setting by attaching a heavy weight to the wrist of the dominant forearm.

Our study had three key findings; First, the border angle where the hand choice probability equals 50% significantly shifted toward the dominant-side after the virtual impairment (i.e., comparison between baseline and pre-test), indicating that the participants had to use their non-dominant side after attaching a wrist weight. Second, the border angle significantly recovered after visual enhancement intervention using the IVR system (i.e., comparison between pre-test and intervention). Third, and most importantly, the border angle was maintained even after the gradual withdrawal of visual amplification (i.e., comparison between pre-test and pos-test), suggesting that the visual enhancement induced a positive and strong effect on the deciding on which hand to use even when the visual amplification was set to 1.0, i.e., no amplification The third point was never reported before, and is promising for the clinical study with stroke patients, as this result indicates that the decision-making process would be biased to use the impaired hand in their daily life after the VR enhanced physical therapy.

Previous studies assert that people suffering from stroke tend to have pre-defined preferences when executing tasks that require manipulation of hands; in most cases, the affected hand is not used (Stoloff et al., 2011; Ballester et al., 2016). The perpetual non-use of a particular hand can harm a patient's quality of life. Indeed, Ballester et al. (2016) claims that stroke patients are sensitive to self-perceived failure; specifically, that the hesitation in using the affected limb is due to a fear of failure and sensitivity about their perceived limitation. In the IVR environment, user can “look around” the artificial world and interact with virtual features or objects. Through iterative visual-motor loops in the brain, the person can have a feeling of controlling the virtual image of their body in such a way that the virtual world would be perceived as a real one. As a result, this technology might support the feeling of embodiment toward the virtual avatar that can be observed from a first-person perspective (Abtahi et al., 2019).

Gonzalez-Franco et al. (2020) explore the impact of motor behavior based on the avatar embodiment. Motor behavior of subjects might be affected when there is discrepancy of the avatar movement that is temporally or spatially deviated from the executed action. In their study, they implement an effect in which the avatar hand drifts either gradually or instantly. Their results show that when there is a mismatch between the real body movement and the virtual avatar, participants try to compensate such a mismatch. This is an example of the “power of the follower effect” which is an active strategy to reduce the conflict of the visuo-proprioceptive feedback coming from spatial mismatch, so users will change their behaviors as much as they can (Gonzalez-Franco et al., 2020).

In this study, it is intriguing that most of participants were not aware of the visual enhancement during the intervention. Within the IVR, the coordinated systems between body representation and vision provides a matching and, based on the findings, the moderate visual enhancement was subconsciously embedded into the cognitive functions of participants' lower-level motor coordination. This enhanced motor performance likely affected the neural pathway of decision-making in the usage of the affected and unaffected upper limbs.

Over the years, many studies have been conducted to investigate the impact of modified visual feedback on body consciousness and behavior (Slater et al., 2008; Burin et al., 2019; Keenaghan et al., 2020; Aoyagi et al., 2021), indicating that sense of agency is affected by the visual feedback. This is because the mismatch between the intended motion and the actual visual feedback directly affects the level of the sense of agency. In our study, we think that the reaching motion within the IVR can contribute to mask the mismatch between the actual upper limb motion and the visually amplified feedback, because the participants do not observe their own upper limb motion in reality. Thus, the participants could feel the higher sense of agency during the visual feedback intervention, resulting in the biased-decision making process to use the impaired upper limb after the removal of the visual amplification.

There can be different reasons why positive visual feedback facilitates the usage of the affected upper limb. Karsh and Eitam (2015) found that the positive judgment of agency over actions encourages the individual to adapt into the continuous changing environment and achieve desired goals, and they discussed that a positive judgement of agency activates the brain's reward system that in turn biases action selection toward actions that were associated with the largest amount of agency. Thus, in the future study, the sense of the agency in the IVR before and after the intervention will be a very interesting point to explore.

Our findings indicated that once the visual enhancement activates a neuronal pathway of using the affected hand in terms of the decision-making process, this neural pathway continues to be active even after the visual enhancement is taken away. Further, this findings was observed even though the intervention (using the enhanced visual feedback) was made available for only a certain period of time. Therefore, our IVR visual enhancement training system could potentially offer a new type of intervention for stroke rehabilitation, incorporating the immersive subjective experience of motor coordination.

Like all other studies, this study also had its limitations. For instance, applying the wrist weight on the dominant hand of some participants encouraged them to manipulate it more as an exercise. Excessive manipulation of the simulated hand with weight does not simulate the features of the paretic limb. In future studies, the hand grip test might be used to determine a suitable weight for individual participants. Secondly, statistics indicate that stroke is more prevalent in people of advanced ages, besides the sense of agency tends to reduce with age (Moore, 2016). A study found that older adults (mean age 78.47 years) were more sensitive to changes in the experimental variables, lag between cursor and mouse movements of 250 or 500 ms turbulence less compared to college students. The possible reason could be college students are more experienced in handling mouse control on computer. Future studies should include a higher percentage of older participants to ensure that the sense of agency suggested by this study is perpetuated to other age groups, specifically the target patient age group (Metcalfe et al., 2010). In summary, the future study will help us understand how the sense of agency is maintained and how motor adaptation can be created when training within the IVR environment.

## Data Availability Statement

The raw data supporting the conclusions of this article will be made available by the authors, without undue reservation.

## Ethics Statement

The experimental protocols in this study were approved by the ethics committees of Tokyo University of Agriculture and Technology (No.191204-3145) and the University of Reading (No.SBS18-19 17). The participants provided written informed consent to participate in the study. Written informed consent was obtained from the individual in Figure 1 for the publication.

## Author Contributions

TK conceived and supervised the study. NS designed the experiment, developed the experimental system, signal processing, and statistical analysis. NS and SA performed the participant experiments. NS, SA, YH, and TK contributed to the discussion of the results. SA and TK wrote the draft of the manuscript. YH, TS, and SY provided critical review of the experimental procedure and analysis. All authors have read and approved the final manuscript.

## Conflict of Interest

The authors declare that the research was conducted in the absence of any commercial or financial relationships that could be construed as a potential conflict of interest.

## References

[B1] Abtahi P. GonzalezFranco M. Ofek E. Steed A. (2019). I'm a Giant: walking in large virtual environments at high speed gains, in Proceedings of the 2019 CHI Conference on Human Factors in Computing Systems (Glasgow), 1–13. 10.1145/3290605.3300752

[B2] Adamovich S. V. Merians A. S. Boian R. Tremaine M. Burdea G. S. Recce M.. (2004). A virtual reality based exercise system for hand rehabilitation post-stroke: transfer to function. Conf. Proc. IEEE Eng. Med. Biol. Soc. 7, 4936–4939. 10.1109/IEMBS.2004.1404364 17271420

[B3] Aoyagi K. Wen W. An Q. Hamasaki S. Yamakawa H. Tamura Y.. (2021). Modified sensory feedback enhances the sense of agency during continuous body movements in virtual reality. Sci. Rep. 11:2553. 10.1038/s41598-021-82154-y 33510374 PMC7844046

[B4] Arbib M. (1981). Perceptual structures and distributed motor control, in Handbook of Physiology, Section I: The Nervous System. 2: Motor Control, ed Brooks V. B. (Baltimore, MD: Williams and Wilkins).

[B5] Ballester B. R. Maier M. San Segundo Mozo R. M. Castaneda V. Duff A. Verschure P. F. (2016). Counteracting learned non-use in chronic stroke patients with reinforcement-induced movement therapy. J. Neuroeng. Rehabil. 13:74. 10.1186/s12984-016-0178-x 27506203 PMC4979116

[B6] Borstad A. L. Crawfis R. Phillips K. Pax Lowes L. Maung D. McPherson R.. (2018). In-home delivery of constraint-induced movement therapy via virtual reality gaming. J. Patient Center. Res. Rev. 5, 6–17. 10.17294/2330-0698.1550 31413992 PMC6664341

[B7] Burdea G. C. (2003). Virtual rehabilitation-benefits and challenges. Methods Inf. Med. 42, 519–523. 10.1055/s-0038-1634378 14654886

[B8] Burin D. Kilteni K. Rabuffetti M. Slater M. Pia L. (2019). Body ownership increases the interference between observed and executed movements. PLoS ONE 14:e0209899. 10.1371/journal.pone.0209899 30605454 PMC6317814

[B9] Dobkin B. H. (2008). Training and exercise to drive poststroke recovery. Nat. Clin. Pract. Neurol. 4, 76–85. 10.1038/ncpneuro0709 18256679 PMC4099052

[B10] Elor A. Kurniawan S. (2020). The ultimate display for physical rehabilitation: a bridging review on immersive virtual reality. Front. Virt. Real. 1:585993. 10.3389/frvir.2020.585993

[B11] Elor A. Kurniawan S. Teodorescu M. (2018a). Towards an immersive virtual reality game for smarter post-stroke rehabilitation, in IEEE International Conference on Smart Computing (SMARTCOMP) (Taormina), 219–225. 10.1109/SMARTCOMP.2018.00094

[B12] Elor A. Powell M. Mahmoodi E. Hawthorne N. Teodorescu M. Kurniawan S. (2020). On shooting stars: comparing CAVE and HMD immersive virtual reality exergaming for adults with mixed ability. ACM Trans. Comput. Healthc. 1, 1–22. 10.1145/3396249

[B13] Elor A. Teodorescu M. Kurniawan S. (2018b). Project star catcher: a novel immersive virtual reality experience for upper limb rehabilitation. ACM Trans. Access. Comput. 11, 1–25. 10.1145/3265755

[B14] Faria-Fortini I. Michaelsen S. M. Cassiano J. G. Teixeira-Salmela L. F. (2011). Upper extremity function in stroke subjects: relationships between the international classification of functioning, disability, and health domains. J. Hand Ther. 24, 257–265. 10.1016/j.jht.2011.01.002 21420279

[B15] Gonzalez-Franco M. Cohn B. Ofek E. Burin D. Maselli A. (2020). The self-avatar follower effect in virtual reality, in IEEE Conference on Virtual Reality and 3D User Interfaces (VR) (Atlanta, GA), 18–25. 10.1109/VR46266.2020.00019

[B16] Jang S. H. Kim Y.-H. Cho S.-H. Lee J.-H. Park J.-W. Kwon Y.-H. (2003). Cortical reorganization induced by task-oriented training in chronic hemiplegic stroke patients. Neuroreport 14, 137–141. 10.1097/00001756-200301200-00025 12544845

[B17] Karsh N. Eitam B. (2015). I control therefore I do: judgments of agency influence action selection. Cognition 138, 122–131. 10.1016/j.cognition.2015.02.002 25724007

[B18] Keenaghan S. Bowles L. Crawfurd G. Thurlbeck S. Kentridge R. W. Cowie D. (2020). My body until proven otherwise: Exploring the time course of the full body illusion. Conscious. Cogn. Neurosci. 78:102882. 10.1016/j.concog.2020.102882 31958664

[B19] Kwakkel G. Veerbeek J. M. van Wegen E. E. Wolf S. L. (2015). Constraint-induced movement therapy after stroke. Lancet Neurol. 14, 224–234. 10.1016/S1474-4422(14)70160-7 25772900 PMC4361809

[B20] Lannin N. A. Horsley S. A. Herbert R. McCluskey A. Cusick A. (2003). Splinting the hand in the functional position after brain impairment: a randomized, controlled trial. Arch. Phys. Med. Rehabil. 84, 297–302. 10.1053/apmr.2003.50031 12601664

[B21] Levin M. F. Kleim J. A. Wolf S. L. (2008). What do motor “recovery” and “compensation” mean in patients following stroke? Neurorehabil. Neural Rep. 23, 313–319. 10.1177/1545968308328727 19118128

[B22] Mang C. S. Campbell K. L. Ross C. J. Boyd L. A. (2013). Promoting neuroplasticity for motor rehabilitation after stroke: considering the effects of aerobic exercise and genetic variation on brain-derived neurotrophic factor. Phys. Ther. 93, 1707–1716. 10.2522/ptj.20130053 23907078 PMC3870490

[B23] Metcalfe J. Eich T. S. Castel A. D. (2010). Metacognition of agency across the lifespan. Cognition 116, 267–282. 10.1016/j.cognition.2010.05.009 20570251

[B24] Moore J. W. (2016). What is the sense of agency and why does it matter? Front. Psychol. 7:1272. 10.3389/fpsyg.2016.01272 27621713 PMC5002400

[B25] Mukherjee D. Patil C. G. (2011). Epidemiology and the global burden of stroke. World Neurosurg. 76(6 Suppl.), 85–90. 10.1016/j.wneu.2011.07.023 22182277

[B26] Nelles G. Jentzen W. Jueptner M. Müller S. Diener H. (2001). Arm training induced brain plasticity in stroke studied with serial positron emission tomography. Neuroimage 13, 1146–1154. 10.1006/nimg.2001.0757 11352620

[B27] Ordahan B. Karahan A. Basaran A. Turkoglu G. Kucuksarac S. Cubukcu M.. (2015). Impact of exercises administered to stroke patients with balance trainer on rehabilitation results: a randomized controlled study. Hippokratia 19:125. 27418760 PMC4938102

[B28] Perez-Marcos D. Chevalley O. Schmidlin T. Garipelli G. Serino A. Vuadens P.. (2017). Increasing upper limb training intensity in chronic stroke using embodied virtual reality: a pilot study. J. Neuroeng. Rehabil. 14:119. 10.1186/s12984-017-0328-9 29149855 PMC5693522

[B29] Rensink M. Schuurmans M. Lindeman E. Hafsteinsdóttir T. (2009). Task-oriented training in rehabilitation after stroke: systematic review. J. Adv. Nurs. 65, 737–754. 10.1111/j.1365-2648.2008.04925.x 19228241

[B30] Slater M. Perez-Marcos D. Ehrsson H. H. Sanchez-Vives M. V. (2008). Towards a digital body: the virtual arm illusion. Front. Hum. Neurosci. 2:6. 10.3389/neuro.09.006.2008 18958207 PMC2572198

[B31] Stoloff R. Taylor J. Xu J. Ridderikhoff A. Ivry R. (2011). Effect of reinforcement history on hand choice in an unconstrained reaching task. Front. Neurosci. 5:41. 10.3389/fnins.2011.00041 21472031 PMC3066466

[B32] Viau A. Feldman A. G. McFadyen B. J. Levin M. F. (2004). Reaching in reality and virtual reality: a comparison of movement kinematics in healthy subjects and in adults with hemiparesis. J. Neuroeng. Rehabil. 1:11. 10.1186/1743-0003-1-11 15679937 PMC546398

